# Sex-Specific Adaptive Strategies of *Populus euphratica* Along Developmental and Canopy Gradients Based on Leaf Trait Networks

**DOI:** 10.3390/plants15121770

**Published:** 2026-06-08

**Authors:** Xiaoli Han, Jie Wang, Xiu Li, Jinlong Zhang, Juntuan Zhai, Zhijun Li

**Affiliations:** 1 Desert Poplar Research Center of Tarim University, Aral 843300, China; lilyan0509@163.com (X.H.); wjie2023418@163.com (J.W.); 15713581176@163.com (X.L.); zhangjl0614@outlook.com (J.Z.); 2 College of Life Science and Technology, Tarim University, Aral 843300, China; 3 Xinjiang Production & Construction Corps Key Laboratory of Protection and Utilization of Biological Resources in Tarim Basin, Aral 843300, China

**Keywords:** *Populus euphratica*, sexual dimorphism, leaf trait network, developmental stage, adaptation strategy

## Abstract

To reveal the variation patterns and differences in the adaptation strategies of leaf functional traits between male and female *Populus euphratica* in an arid desert environment, this study evaluated the effects of sex, developmental stage, and their interaction on 31 leaf traits using variance partitioning and trait network analysis. Furthermore, we analyzed the topological characteristics of the trait networks across two dimensions: developmental stage and vertical canopy gradient. The results indicated that sex moderately explained the variation in leaf nutrient characteristics (N and K) and physiological resistance indicators (Pro). Meanwhile, developmental stage largely accounted for variations in traits such as leaf dry weight, leaf width, specific leaf area, and photosynthetic physiology. The interaction between sex and developmental stage significantly influenced leaf anatomical structures and water-use strategies. Leaf trait network analysis revealed that during development, the male network exhibited higher connectivity and shorter average path lengths, with its core traits shifting from photosynthetic physiological indicators to nutrient and water transport characteristics; female plants exhibited higher network modularity during key developmental stages, with core nodes concentrated on leaf area, biomass, and structural traits. Along the vertical canopy gradient, the male leaf trait network showed pronounced topological reorganization in the mid-to-upper layers, suggesting a stronger capacity to respond to environmental fluctuations. Conversely, the core hubs of the female leaf trait network shifted from morphogenesis toward a synergy between structure and metabolism, which may be associated with maintaining system stability at different canopy heights. These findings suggest that female and male *P. euphratica* may adopt “conservative” and “acquisitive” ecological adaptation strategies, respectively, potentially leading to differentiated patterns of trait variation and coordination. This study provides a theoretical basis for understanding the potential ecological adaptation mechanisms and evolutionary strategies underlying sexual dimorphism in desert plants.

## 1. Introduction

Global climate change has intensified the frequency and severity of droughts, profoundly threatening ecosystem stability [1]. Arid regions account for approximately one-third of the global land surface and are crucial for maintaining global ecological balance, as well as the carbon and water cycles [2,3]. However, rising temperatures, shifting precipitation patterns, and the increased frequency of extreme weather events driven by climate change not only alter plant distribution ranges and cause a decline in community diversity [4,5] but also significantly impair plant morphological and physiological functions, leading to growth inhibition and even mortality [6]. To survive and reproduce in such harsh environments, plants in arid regions have evolved a variety of strategies at the morphological, structural, physiological, and molecular levels to minimize water loss or to adapt to growth under water-deficient conditions [7].

Leaf traits are critical indicators of plants’ environmental adaptation strategies, reflecting their capacity for resource acquisition and utilization, as well as their resistance and resilience to environmental stress [8,9]. Throughout long-term evolution, plants have developed diverse combinations of functional traits—encompassing leaf phenotypes, anatomical structures, physiological metabolism, and resource allocation—to cope with selective pressures [10]. These traits do not exist in isolation; rather, they are both independent and interrelated, interacting through coordination, trade-offs, coupling, and decoupling to form a complex trait system [11,12,13,14,15]. This shift in analytical perspective from single to multidimensional traits reveals that plants do not adapt to environments through the extreme expression of a single trait, but rather achieve optimal overall performance through coordinated relationships among traits. Therefore, unraveling the correlation patterns and network topological features of multidimensional traits has become a core pathway for understanding the overall adaptation strategies, niche differentiation, and evolutionary logic of plants. Particularly in dioecious plants, the integration and coordinated regulation of multidimensional traits are especially pronounced, driven by differential reproductive investment, the integration and coordinated regulation of multidimensional traits are especially pronounced.

There are approximately 6% of angiosperm species (14,620 out of 240,000) are dioecious, which constitute an essential component of terrestrial ecosystems and play a pivotal role in maintaining biodiversity [16]. Driven by differential reproductive resource allocation, male and female individuals exhibit significant sexual dimorphism in morphological, structural, and physiological traits [17,18,19]. Morphologically and anatomically, females of *Podocarpus macrophyllus* possess significantly larger leaf areas than males, whereas males meet their reproductive resource demands by increasing branching and leaf numbers [20]. Similarly, females of *Cercidiphyllum japonicum* exhibit a higher SLA to enhance light capture [21,22], while males of *Hippophae rhamnoides* reduce resource consumption through a lower SLA [23,24]. Males of species such as *Broussonetia papyrifera* and *Sabina vulgaris* optimize leaf anatomical structures—such as the palisade-to-spongy tissue ratio, epidermal thickness, and vascular tissues—to enhance water storage and utilization [25,26]. At the physiological level, these sexual differences primarily reflect a trade-off between resource acquisition and resistance maintenance. To meet the high energy demands of fruit and seed development, females of species like *Salix arctica* and *H. rhamnoides* typically exhibit higher *Pn* and metabolite accumulation capacities [27]. Conversely, under drought or high-temperature stress, males often demonstrate superior WUE and stomatal regulation, which enhance water retention and survival [28]. Furthermore, sexual differences exist in osmoregulatory patterns; females often strengthen their osmoregulatory capacity by accumulating higher levels of stress-related compounds such as Pro and MDA [29].

Although existing studies have extensively explored the differences between male and female plants in individual traits, they have predominantly focused on localized trait relationships, such as functional axes or the Leaf Economics Spectrum. Limited attention has been paid to the coordinated variation patterns among multiple traits, which limits a holistic understanding of the mechanisms by which sexual differences influence evolution and environmental adaptation. Recently, Plant Trait Networks (PTNs) have emerged as a novel research framework for deciphering complex inter-trait associations [30,31,32,33]. Compared to traditional correlation analyses, PTNs utilize quantitative network structural parameters to not only characterize systemic properties but also identify pivotal traits [34,35]. Through the construction of PTNs, adaptation strategies across different sexes, species, or communities can be compared at a systemic level, offering a fresh perspective for research in plant functional ecology [36,37,38].

All species within the genus *Populus* (Salicaceae) are dioecious, and sex-specific differences in response to environmental stress have been observed in Salicaceae plants such as *P. cathayana*, *P. tremuloides*, and *Salix arctica* [39,40,41]. As a foundation species in arid desert ecosystems, *P. euphratica* is an ideal model for studying sex-specific adaptation strategies in plants. Previous studies have shown that female of *P. euphratica* exhibit stronger stress resistance by reinforcing leaf anatomical structures. This is evidenced by a significant increase in leaf and palisade tissue thickness, as well as the optimization of midrib xylem development and vascular bundle proportion, which ensure effective water transport and robust mechanical support for them in extreme habitats. In contrast, males possess an advantage in regulating physiological homeostasis regulation; under drought stress, they maintain stable photosynthetic efficiency by maintaining higher levels of chlorophyll synthesis and stable leaf water potentials [42].

However, existing research has mostly focused on the static comparison of individual traits, which is insufficient to elucidate how *P. euphratica* constructs its overall defense and growth patterns through synergistic variations and trade-offs among multiple traits. To overcome the limitations of traditional single-trait analyses, this study applies the PTNs approach. This method quantifies the complex interactions, modular characteristics, and core hub traits within trait networks from multiple perspectives, thereby comprehensively evaluating internal resource allocation and functional coordination in plants. Building upon this framework, we systematically measured 31 structural and functional leaf traits in female and male *P. euphratica* plants. By integrating these data with PTN analysis, this study aims to address the following three core scientific questions in depth: (1) What are the effects of sex, developmental stage, and their interactions on leaf trait variation in *P. euphratica*? (2) How do male and female *P. euphratica* plants achieve different trade-off strategies between resource utilization and defense during development through structural differences in leaf trait networks (e.g., modular configuration and hub trait transitions)? (3) In response to microenvironmental variations along the vertical canopy gradient, how does the coordinated regulation of leaf trait networks drive the spatial adaptation strategies of male and female trees?

## 2. Result

### 2.1. Relative Contributions of Sex and Developmental Stage to Leaf Trait Variation and Their Interactive Effects

**Figure 1:**
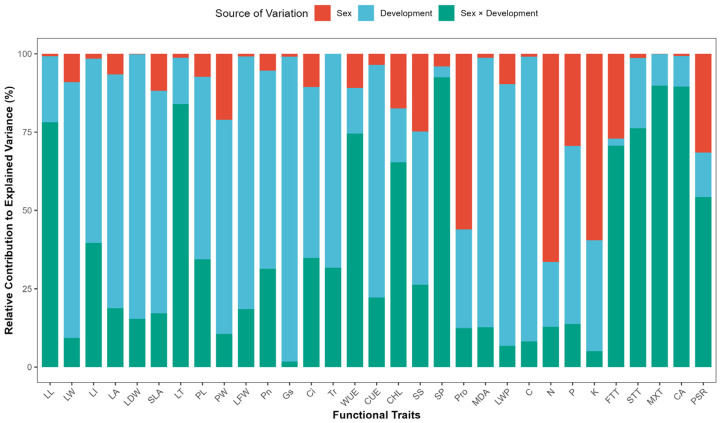
Two-way interaction effects of leaf traits in *P. euphratica*.

Using variance partitioning analysis, we systematically evaluated the relative contributions of sex, developmental stage, and their interaction to the variation in 31 heteromorphic leaf phenotypic traits of *P. euphratica* (Figure 1). Results indicated that variations in total nitrogen (N: 66.46%) and total potassium (K: 59.54%) were primarily driven by the sex effect, and proline (Pro: 56.02%) also exhibited strong sex specificity. Developmental stage drove the variation in multiple traits. Specifically, leaf dry weight (LDW: 84.51%), leaf width (LW: 81.65%), leaf fresh weight (LFW: 80.65%), and specific leaf area (SLA: 71.04%) showed high dependence on the developmental stage. Similarly, stomatal conductance (*Gs*: 97.34%), transpiration rate (*Tr*: 68.26%), and net photosynthetic rate (*Pn*: 63.27%) were also mainly regulated by the developmental stage. In addition, carbon content (C: 91.00%) and water stress indicators, such as malondialdehyde (MDA: 86.07%) and leaf water potential (LWP: 83.61%), exhibited significant variation across developmental stages.

The interaction between sex and developmental stage had significant effects on certain anatomical structures and physiological indices. Specifically, leaf thickness (LT: 83.95%), leaf length (LL: 78.14%), and anatomical traits related to the midrib, lateral veins, and mechanical tissues (MXT: 89.81%; CA: 89.57%; STT: 76.31%) were primarily driven by this interaction. At the physiological level, chlorophyll content (CHL: 65.34%) and water use efficiency (WUE: 74.49%) also exhibited a significant pattern of synergistic regulation.

### 2.2. Differences in the Topological Characteristic Parameters of Leaf Trait Networks Between Male and Female Plants at Different Developmental Stages

**Figure 2:**
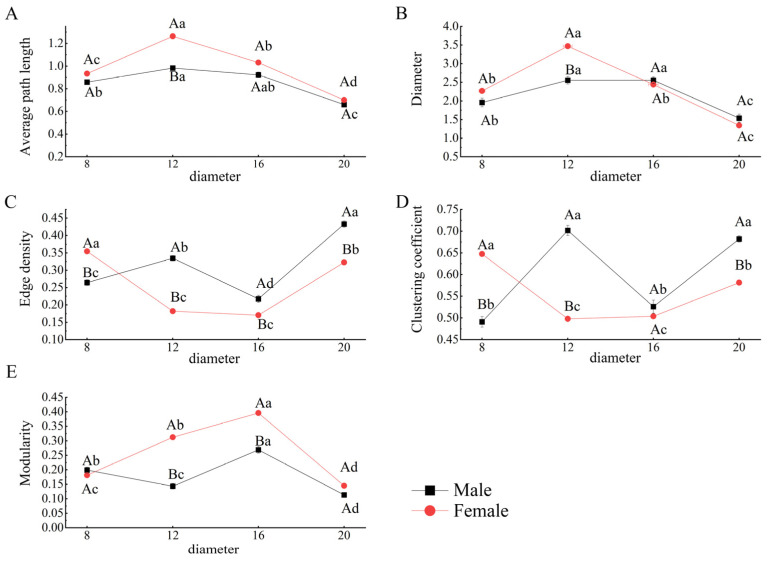
Characteristic differences in leaf trait networks of male and female *P. euphratica* at different developmental stages. (**A**): Average path length; (**B**): Diameter; (**C**): Edge density; (**D**): Clustering coefficient; (**E**): Modularity.

Comparison of network topological parameters between male and female *P. euphratica* plants across developmental stages revealed that, during the early stage (diameter class 8), edge density and clustering coefficient in females were significantly higher than in males, indicating tighter synergistic trait associations at this stage. However, during the middle stage (diameter classes 12 and 16), both edge density and clustering coefficient were higher in males. By the late stage (diameter class 20), edge density and clustering coefficient in males remained significantly higher than in females (Figure 2). In addition, average path length and network diameter in males showed a unimodal trend during development (reaching peaks at diameter classes 12 and 16, respectively, and declining to their minimum at diameter class 20). Similarly, average path length, network diameter, and modularity in females also exhibited a unimodal pattern, with peaks occurring between diameter classes 12 and 16.

### 2.3. Differentiation of Key Nodes and Regulatory Strategies in Leaf Trait Networks of Male and Female Plants at Different Developmental Stages

**Figure 3:**
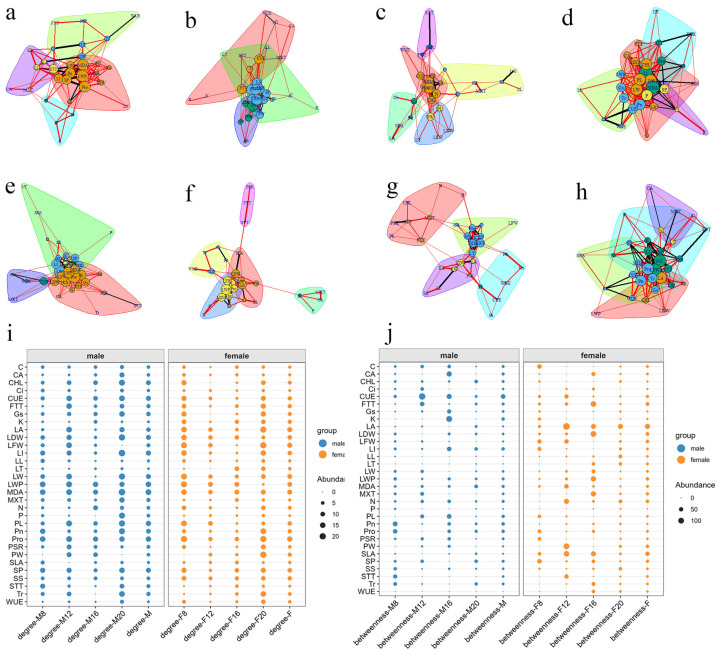
(**a**–**h**): Leaf trait networks of male and female *P. euphratica* at different developmental stages; (**a**): f-8, (**b**): f-12, (**c**): f-16, (**d**): f-20, (**e**): m-8, (**f**): m-12, (**g**): m-16, (**h**): m-20; (**i**,**j**): Network node parameters for leaf traits of male and female *P. euphratica* at different developmental stages; (**i**) is the degree parameter, and (**j**) is the betweenness parameter. Blue circles indicate male trees, whereas red circles indicate female trees.

In the leaf trait networks, trait centrality exhibited significant sex differences and developmental stage heterogeneity (Figure 3). Traits with high betweenness centrality primarily connect different functional modules, whereas traits with high degree centrality maintain tight associations with a greater number of traits. Analysis of network node characteristics across different diameter classes indicated distinct differences in key traits between males and females. In the early developmental stage (diameter class 8), *Pn* and *Tr* in males exhibited extremely high betweenness centrality, while LW and PL showed high degree centrality. As the diameter class increased (classes 16–20), the key traits in the male network shifted to N, CHL, and structural traits such as CA.

Unlike males, LA, LDW, and LFW maintained a high degree of betweenness centrality across various developmental stages in the female network. During the critical developmental periods of diameter classes 12 and 16, the high betweenness of SLA suggested that females might improve resource use efficiency by optimizing leaf investment per unit area. At diameter class 20, LA and LL remained the key traits in the network. In summary, the key traits in the male network were primarily photosynthetic, physiological, and vessel-related traits, whereas those in females focused on leaf morphology and biomass-related traits.

### 2.4. Variation Patterns of Topological Characteristics of Leaf Trait Networks in Male and Female Plants Along Tree Height

**Figure 4:**
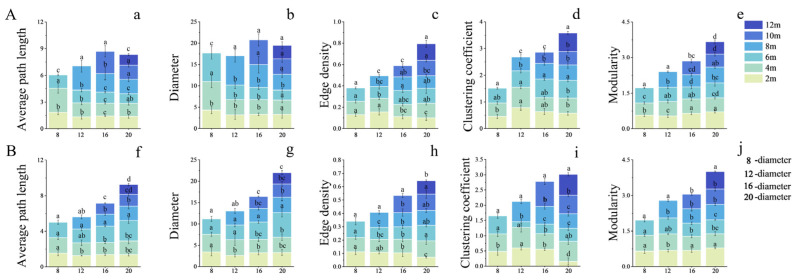
Variations in leaf trait network parameters along the vertical tree height gradient in female (**A**) and male (**B**) *P. euphratica*. (**a**,**f**): Average path length, (**b**,**g**): Diameter, (**c**,**h**): Edge density, (**d**,**i**): Clustering coefficient, (**e**,**j**): Modularity. The letters a, b, c, and d in the figure indicate the results of the multiple comparison test for significance.

The network characteristics of leaf traits in males and females of *P. euphratica* exhibited significant stage-dependent differences as canopy height increased (Figure 4). At diameter class 8, the clustering coefficient in the lower canopy (2 m) of males was significantly lower than that in the middle canopy (4–6 m). Across diameter classes 12–20, leaf trait network parameters exhibited regular changes with increasing canopy height. The average path length and network diameter were at their maximum in the middle and upper canopies (the 6–8 m interval), while the clustering coefficient was at its minimum within the same height range (Figure 4B).

In contrast, the response of leaf trait network parameters in females to the vertical gradient was more continuous. At diameter class 8, the clustering coefficient and network diameter of the middle canopy (4 m) reached their maximum values. For diameter classes 12 and 20, the average path length and network diameter reached their maximum values in the upper canopy (8–10 m), whereas edge density and clustering coefficient remained at high levels in the lower canopy (2 m) (Figure 4A). In conclusion, the leaf trait networks of males demonstrated significant structural variations within the middle and upper canopy layers (4–8 m). In contrast, the networks of females remained relatively stable along the vertical gradient, exhibiting a higher degree of modularity in the lower canopy (2 m).

### 2.5. Variation Patterns of Key Node Characteristics of Leaf Trait Networks in Male and Female Plants Along Tree Height

**Figure 5:**
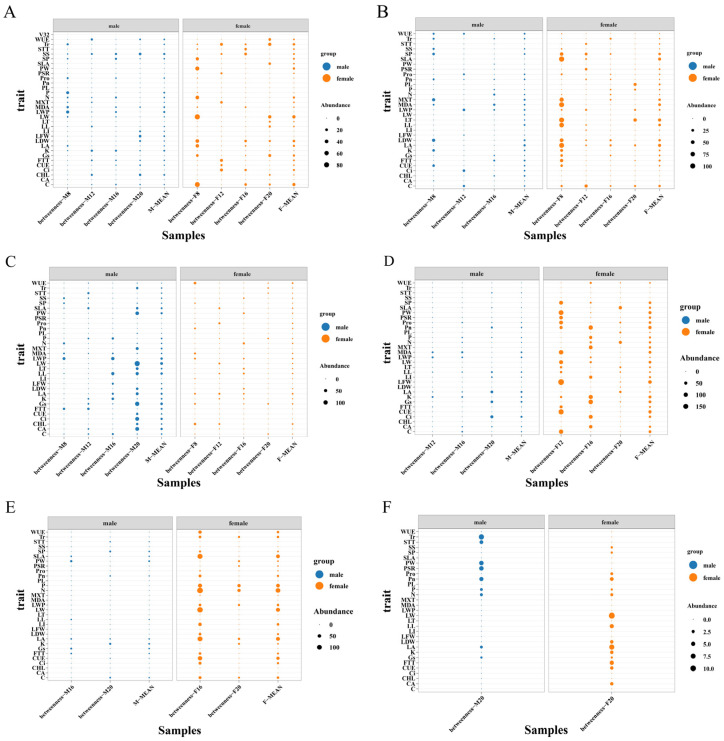
Betweenness centrality of network nodes for leaf traits in male and female *P. euphratica* at different tree heights ((**A**–**F**): 2 m, 4 m, 6 m, 8 m, 10 m, 12 m).

Comparison of leaf trait network structural characteristics between males and females of *P. euphratica* across vertical heights revealed that network topological roles of key traits exhibited significant sexual dimorphism (Figure 5 and Figure S1). In female networks, traits with high betweenness centrality primarily included FTT and LW, while key traits in male networks were primarily LWP and carbon–nitrogen metabolism-related traits. Among stress resistance-related traits, Pro and SP acted as primary nodes in females, whereas MDA was the primary node in males.

At the spatial scale, core nodes of the leaf trait networks showed obvious gradient differences with height. In the lower canopy (Figure S1A and Figure 5A), the key traits of male networks gradually shifted from nutrient-related traits to K and SS during development, and water regulation-related traits shifted from LWP to WUE. For females, early key traits mainly included LA and LW, while mid-to-late stage key traits mainly included palisade tissue structure and C metabolism-related traits. In the middle canopy (Figure S1B,C and Figure 5B,C), sexual differences became more significant with increasing height. At a height of 4 m, male key traits shifted from CA to traits such as MDA and Pro; females gradually shifted from SLA to traits centered on LDW and LT. At a height of 6 m, male key traits mainly included anatomical structure and water transport-related traits, while female traits mainly included osmoregulatory substances and nutrient metabolism-related traits.

In the upper canopy (Figure S1D,E and Figure 5D,E), male key traits were mainly concentrated in ion balance, water status, and oxidative defense-related traits; females mainly exhibited synergistic changes among biomass, stomatal regulation, and nutrient-related traits, with traits such as SLA and phosphorus content showing higher centrality. In the top canopy (Figure S1F and Figure 5F), the differences in key network nodes between males and females were even more distinct. The central traits of males were mainly concentrated on indicators such as *Tr*, *Gs*, LA, and palisade-to-spongy tissue ratio, whereas females exhibited a stronger synergistic relationship among N, SS, and LW. In summary, along the vertical gradient, the core network nodes of males were mainly concentrated in physiological indicators such as water status and gas exchange, while females exhibited a synergistic association between morphological structure and physiological metabolism traits.

## 3. Discussion

### 3.1. Driving Mechanisms of Ontogeny and Sexual Dimorphism on Leaf Trait Variation

Phenotypic plasticity is central to plant adaptation to extremely heterogeneous environments [43]. This study found that the variation in leaf traits of *P. euphratica* exhibited significant functional modularity, which is primarily driven by the differential effects of ontogeny and sexual dimorphism. Ontogeny is the core factor driving the transition of growth investment and resource acquisition patterns in *P. euphratica*. As trees develop from the juvenile to the mature stage, the transition of heterophyllous leaves reflects an optimization strategy for resource utilization [44]. In early development, leaves ensure the stability of core metabolism by maintaining lower levels of variation; as the canopy expands, leaves undergo significant adjustments in biomass accumulation (e.g., LDW, LFW) and photosynthetic physiology (e.g., *Gs*, *Pn*), leading to a substantial increase in efficiency such as WUE [45,46]. This dynamic reshaping across ontogenetic stages allows *P. euphratica* to achieve a life-history strategy transition at the individual level, shifting from ensuring basic survival during the juvenile stage to meeting the demands of rapid growth and reproduction in the adult canopy.

In contrast, sexual dimorphism plays a dominant role in nutrient allocation and the maintenance of physiological resistance, reflecting differences in resource trade-offs between males and females in response to reproductive costs. Males exhibit higher physiological plasticity and tend to adopt an “acquisitive” strategy [19,47], prioritizing investment in physiological metabolism through the flexible regulation of nutrient uptake (e.g., N, K) and energy conversion to respond rapidly to short-term environmental fluctuations. Conversely, females adopt a “conservative” strategy [48], maintaining high stability in structural tissues (e.g., LT) and carbon balance (e.g., C), while prioritizing structural defense and stomatal regulation to ensure long-term reproductive security and a stable energy supply.

Notably, the interaction between sex and ontogeny profoundly regulates leaf anatomical structures (e.g., STT, MXT, CA), indicating that sexual dimorphism in *P. euphratica* possesses a clear ontogenetic contingency. To gain a deeper understanding of the systemic dynamics of this complex relationship, this study explored the trait networks of males and females across different developmental stages and canopy heights. Males constructed a trait network with high connectivity and short path lengths during the mature stage, a structure that facilitates rapid synergy of physiological signals and resources to support high-intensity energy expenditure during the flowering period. In contrast, females exhibited high modularity during key developmental stages; by enhancing the relative independence of functional units, this modularity helps reduce interference between different physiological processes, thereby maintaining overall system stability in the context of high reproductive investment [49]. In summary, *P. euphratica* constructs a multi-dimensional adaptation strategy by combining ontogeny-driven morphological remodeling with sexual dimorphism-driven physiological regulation [50].

### 3.2. Differences in Leaf Trait Networks and Environmental Adaptation Strategies of Male and Female Across Developmental Stages

During the early stages of growth, trees tend to allocate more resources to structural development, which may lead to an initial decline in resource utilization efficiency [51]. As the canopy and root systems expand during ontogeny, trees gradually enhance their light-harvesting capacity and their ability to absorb water and nutrients, thereby promoting growth and more efficient resource utilization [52]. In this study, the resource utilization capacity of *P. euphratica* followed a trend of “initial decrease followed by an increase” with increasing diameter classes, reflecting the dynamic optimization of resource allocation during development. Particularly upon entering the mature stage (e.g., diameter class 20), the trait network exhibited shorter average path lengths and lower modularity. Such topological optimization suggests that the coordination efficiency among traits reached its peak, thereby significantly enhancing the resource integration capacity of the overall system.

Despite the consistency in general resource utilization trends, the developmental trajectories of the trait networks revealed significant sex-specific strategy differences. Males exhibited continuously enhanced stress resistance regulation and resource acquisition capabilities [42,53]. In early development, the network core was dominated by LWP and *Pn*, ensuring basic water balance and carbon fixation. As development progressed, males strengthened the synergy between mineral nutrition and osmotic regulation by increasing the connectivity of indicators such as K and Pro within the network. By the mature stage, the high integration of MDA and leaf shape indicators further improved the oxidative homeostasis of the system. This network reorganization mechanism enables males to maintain efficient physiological responses, aligning with their “acquisitive” and highly stress-resistant niche characteristics.

Compared to the sustained enhancement of stress resistance in males, the trait coordination in females transitioned from stress resistance regulation toward structural growth and morphogenesis. Early-stage females similarly utilized osmotic regulation traits such as Pro and LWP as the network core to cope with environmental stress. However, upon entering the mid-developmental stage, the centrality of structural traits (e.g., PL, LDW, and FTT) in the network increased significantly. By the mature stage, biomass and light-reception area indicators such as LFW and LA occupied the network hubs. This stage-specific functional transition reflects the reproductive trade-off strategy of females [54]: to meet the high energy demands of seed development and reproductive structures, females likely prioritize morphogenesis and energy reserves in later developmental stages. This shift from stress resistance to biomass accumulation represents an adaptive compromise reached by females to balance individual survival with reproductive success.

### 3.3. Differences in Leaf Trait Networks and Environmental Adaptation Strategies of Male and Female Across Vertical Tree Heights

Due to significant variations in microenvironmental factors such as light intensity, transpiration, gas exchange, and wind velocity along vertical gradients, plants often optimize their overall resource acquisition and utilization efficiency by developing specific leaf functional traits at different tree heights [55,56]. This study found that with increasing tree height, females tend to utilize anatomical structures (e.g., FTT) and morphological traits (e.g., LW, SLA) as network cores, while males employ physiological metabolic indicators (e.g., LWP, MDA) as regulatory hubs, reflecting inherent resource trade-off differences between the sexes in vertical space utilization.

On a spatial scale, these strategic differences exhibit distinct gradient characteristics. In the lower-to-middle canopy (2–6 m), females prioritize the construction of photosynthetic structures by expanding LA and LW to enhance their ability to capture limited light resources. In contrast, males shift rapidly from nutrient synergy to a regulatory path centered on WUE, demonstrating high sensitivity to water fluctuations. As height increases to the upper and top canopy (8–12 m), leaf trait networks tend toward functional clustering, and sexual dimorphism becomes significantly more pronounced. At 12 m, the male network primarily focuses on traits related to stomatal regulation and water transport (e.g., CA) to cope with hydraulic limitations at the canopy top. Meanwhile, females construct a multidimensional synergistic regulation mode through the tight coupling of N content and SS, thereby maintaining stable physiological functions and long-term adaptability in a high-stress environments.

These differences indicate that male and female *P. euphratica* adopt distinct strategies when responding to vertical spatial pressure. The male strategy emphasizes rapid resource integration and environmental response efficiency, whereas the female strategy favors network stability and functional redundancy. This reflects a highly buffered adaptive system constructed by females to support reproductive costs [57]. Such divergent strategies manifest the distinct physiological trade-offs and ecological adaptation pathways of male and female trees in arid environments. Although microenvironmental measurements along the vertical canopy profile were not conducted in this study, the presence of significant vertical environmental gradients within forest canopies is well-documented. Previous research has demonstrated that light intensity attenuates exponentially from the upper canopy downwards [58], whereas vapor pressure deficit and wind speed typically increase with height. Therefore, we speculate that the vertical trait variations observed in this study are likely the adaptive responses of male and female *P. euphratica* to the predictable microenvironmental heterogeneity at different canopy heights [59].

## 4. Materials and Methods

### 4.1. Overview of the Study Area

The study area is located in an artificial *P. euphratica* forest on the northwestern edge of the Tarim Basin, Xinjiang (180.6 hm^2^; E 81°17′56.52″, N 40°32′36.90″; elevation 980 m). The forest comprises both female and male *P. euphratica* individuals across different diameter classes. This artificial *P. euphratica* forest was established in 1990 and has been managed as a long-term experimental site. Management practices primarily consisted of artificial irrigation, regular weeding, and pest control, while grazing and other human disturbances were strictly restricted to maintain stable stand growth. The study area is characterized by a hot and dry climate, with an average annual precipitation of approximately 50 mm, a mean annual temperature of 10.8 °C, potential evaporation exceeding 1900 mm, and an average annual sunshine duration of 2900 h. It belongs to a typical temperate continental extremely arid desert climate zone. The habitat conditions were as follows: soil water content was 27.53%, daily mean air temperature was 31.09 °C, daily mean air humidity was 36.78%, and daily mean light intensity was 11,869.78 lx. Meteorological data were obtained from the long-term average records of WorldClim. Air temperature, relative humidity, and light intensity were measured using a portable environmental monitoring device, from which daily mean values were calculated. Soil water content was determined using a soil moisture meter.

### 4.2. Experimental Design and Sampling

To minimize the influence of environmental factors, female and male *P. euphratica* individuals from different diameter classes exhibiting uniform growth were selected under consistent habitat conditions. All sampled trees were healthy without visible signs of pests, diseases, or mechanical damage. The sample trees were categorized into diameter classes using a 4 cm interval. Specifically, three female and three male trees were selected from each of the 8, 12, 16, and 20 cm diameter classes, resulting a total of 24 sample trees (Table 1). A measuring tape and a laser hypsometer were used to measure the diameter at breast height (DBH) and tree height (Haglöf, Sweden). Starting from the base of the main stem, sampling points were established at 2 m intervals at 2, 4, 6, 8, 10, and 12 m, representing different canopy heights. Considering the effects of light and other factors on leaf growth, three current-year branches were collected from each of the four cardinal directions at each sampling point. Leaves at the fourth node from the base toward the tip of each branch were selected as samples for measuring the morphological and anatomical traits of heteromorphic leaves.

#### 4.2.1. Measurement of Morphological Traits of Heteromorphic Leaves

During the sampling phase, four diameter classes (8, 12, 16, and 20) were established for both female and male *P. euphratica* individuals, with 3 sample trees selected per diameter class. Based on differences in tree height, various canopy sampling heights were designated. At each sampling point, 3 current-year branches were collected from the four cardinal directions. The leaf at the 4th node (from the base toward the apex) of each branch was consistently selected as the representative sample. A total of 1296 leaves were collected from both trees. A portable leaf area meter LI-3000C (LI-COR, USA) was used to measure the LL, LW, and LA of the samples. The LI was calculated as the ratio of LL to LW. LT and PT were measured using a digital vernier caliper, while PL was determined using a ruler. The fresh weight of leaves without petioles (LFW) was determined using an electronic balance with a precision of 0.0001 g. Subsequently, the leaves were placed in a constant-temperature oven at 105 °C for 10 min to deactivate enzymes, and then dried at 80 °C until reaching a constant mass. The leaf dry weight (LDW) was measured to calculate the specific leaf area (SLA). SLA was defined as the ratio of LA to LDW.

#### 4.2.2. Measurement of Anatomical Structural Traits of Heteromorphic Leaves

The leaves used for determining the anatomical structure were collected following the same protocol as the morphological measurements. Transverse sections were taken from the widest part of each sample leaf and fixed in a formalin–alcohol–acetic acid (FAA) solution. Samples were prepared using the standard paraffin sectioning method, with a section thickness of 8 μm. The sections were double-stained with safranin and fast green, and then mounted with neutral resin. The following parameters were observed and measured under a Leica microscope (Leica Microsystems GmbH, Wetzlar, HE, Germany): FTT, STT, MXT, and CA. The PSR was calculated as the ratio of FTT to STT. For each leaf, 5 fields of view were observed, with 20 measurements taken in each field. The mean value from the 5 fields of view was used as the final value for each anatomical trait.

#### 4.2.3. Determination of Physiological Indicators of Heteromorphic Leaves

In June-July 2020, leaf photosynthetic parameters were measured using a LI-6400 portable photosynthesis system (LI-COR, Inc., Lincoln, NE, USA). Net photosynthetic rate (*Pn*), stomatal conductance (*Gs*), intercellular CO_2_ concentration (*Ci*), and transpiration rate (*Tr*) were measured under clear and cloudless conditions between 09:00 and 11:30 a.m., avoiding midday depression. During the measurements, light conditions were stable, and no rainfall or strong wind interference occurred. At each sampling site, the leaf at the fourth node (from the base toward the apex) of the sampled branch was selected. For each treatment, 12 leaves were measured in three replicates. The measurement conditions were set to: photosynthetic photon flux density of 1200 μmol m^−2^ s^−1^, leaf temperature of 25 °C, and ambient CO_2_ concentration of 400 μmol mol^−1^. Instantaneous water use efficiency (WUE) was calculated as the ratio of *Pn* to *Tr* (WUE = *Pn*/*Tr*) [60].

The leaf at the fourth node (from the base toward the apex) of each branch was selected, and the LWP was measured using a portable plant water potential pressure chamber (Model 600-EXP; PMS Instrument Company, Albany, OR, USA). Measurements were conducted at pre-dawn and completed within 2 h. The selected leaves were excised and measured immediately. For each treatment, 12 leaves were measured, and the mean value was calculated.

After the measurement of leaf morphological traits, the samples were immediately rinsed with distilled water, deactivated at 105 °C for 10 min, and then dried in an oven at 60 °C for 48 h until reaching a constant weight. The dried samples were ground and passed through a 100-mesh sieve. Plant samples for carbon isotope analysis were prepared using a glass vacuum system. The combustion furnace was maintained at 1000 °C. After evacuation, oxygen was introduced into the system. The porcelain spoon containing the sample was placed in the combustion tube and combusted in the high-temperature zone for 2 min. The CO_2_ gas was then collected, purified by freezing, and analyzed for carbon isotope composition using an isotope mass spectrometer (Finnigan MAT, San Jose, CA, USA).

Composite leaf samples from the fourth node of current-year branches within the same canopy layer were selected. Leaf physiological indicators were determined using the micro-method. Assay kits produced by Suzhou Comin Biotechnology Co., Ltd. (Suzhou, China) were used according to the manufacturer’s instructions to determine the proline content (PROC), malondialdehyde content (MDAC), soluble sugar content (SSC), and soluble protein content (SPC).

### 4.3. Statistical Analysis

**Figure A1:**
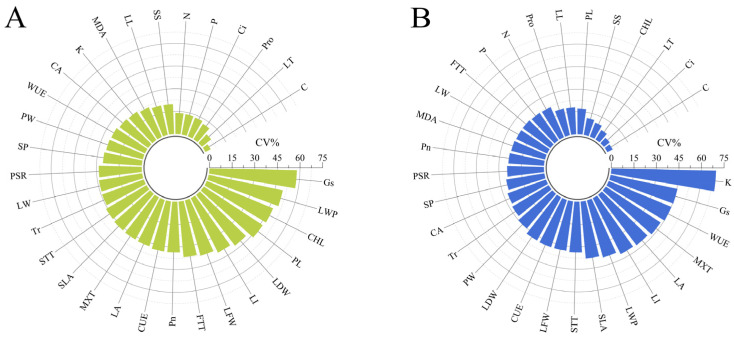
Variation analysis of leaf traits in female and male *P. euphratica*. (**A**): Female, (**B**): Male.

A two-way analysis of variance (ANOVA) was employed to evaluate the effects of sex, developmental stage, and their interaction on 31 leaf traits. The variance partitioning analysis was conducted within a fixed-effects two-way ANOVA framework, where each trait was modeled separately. The model included three fixed effects: sex, developmental stage, and their interaction. The model was expressed as *Y_ijk_* = *μ* + *S_i_* + *D_j_* + (*SD*)*_ij_* + *ε_ijk_*. Here, *Y_ijk_* represents the trait value, μ is the overall mean, *S_i_* represents the fixed effect of sex, *D_j_* represents the fixed effect of developmental stage, (*SD*)*_ij_* represents the interaction term, and *ε_ijk_* is the residual term. Hierarchical partitioning was performed using the glmm.hp package to quantitatively determine the contribution of each factor to the total explained variance. Additionally, the coefficient of variation (CV) for each trait was calculated. A higher CV generally reflects greater regulatory flexibility of a specific sex in a functional dimension, whereas a lower CV indicates trait conservatism or the ability to maintain homeostasis. The coefficients of variation (CV) for various traits of female and male *P. euphratica*, along with their ecological implications, are presented in Appendix A.1 (Figure A1).

**Figure A2:**
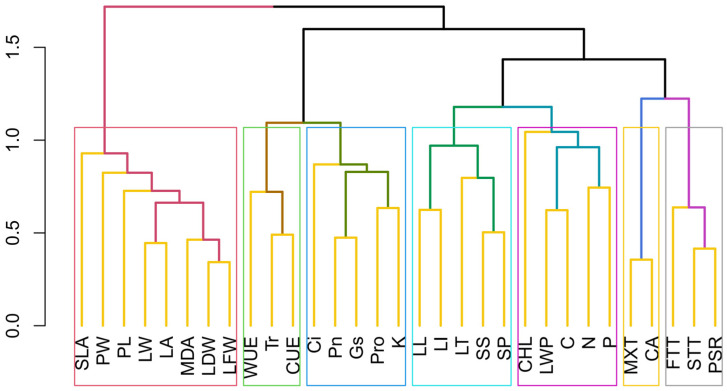
Hierarchical cluster analysis of leaf traits.

**Figure A3:**
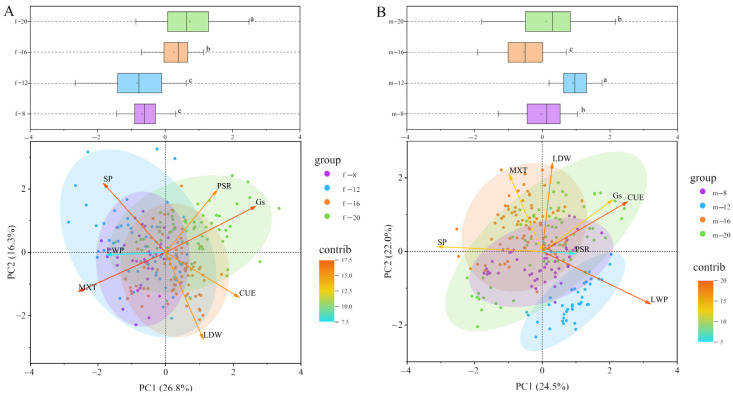
Principal component analysis of leaf traits in female and male *P. euphratica* at different developmental stages. (**A**): Female, (**B**): Male.

To identify representative indicators from multi-dimensional traits, hierarchical cluster analysis (HCA) was used to classify traits into core functional modules. Based on the Pearson correlation matrix, 1-|r| was used as the distance metric, and Ward’s D^2^ method was applied to divide the 31 traits into 7 core functional modules. The trait with the highest mean absolute correlation within each module was selected as the core indicator (Appendix A.2; Figure A2). Subsequently, principal component analysis (PCA) was conducted to reveal the differentiation patterns of male and female plants in multi-dimensional trait space, and biplots were used to systematically display the coupling relationships among key traits, sex, and developmental stage. The explained variance, variable loadings, and ecological implications of each principal component are presented in Appendix A.3 and Figure A3.

Leaf trait networks (LTNs) were constructed for male and female *P. euphratica* plants at different developmental stages to reveal differences in trait association patterns during growth. In the LTN, plant traits are represented as nodes, and associations between them are represented as edges [61]. First, a correlation matrix was calculated using Pearson correlation coefficients; the absolute value, |r|, represented the strength of associations. To avoid spurious relationships, only significant associations at the *p* < 0.05 level were retained: significant correlations were assigned a value of 1, whereas non-significant correlations were assigned a value of 0. Accordingly, an adjacency matrix A = [a_i,j_] was constructed, where a_i,j_ ∈ [0, 1]. Thus, the LTN indicated the presence or absence of correlations. Additionally, the absolute values of correlation coefficients were used to weight the edges between any pair of leaf traits [34], and the LTN was finally visualized.

Five global network parameters and two node-level parameters were selected to describe the network characteristics. Network diameter refers to the maximum shortest path length between any two connected nodes. Average path length represents the mean shortest path length among all nodes in the network. Edge density is defined as the ratio of the total number of observed edges to the maximum possible number of edges [62]. Modularity describes the degree of separation among subnetworks or modules, with traits of similar functions tending to organize into the same module [63]. The average clustering coefficient is defined as the mean clustering coefficient of all traits in the leaf trait network. A higher value indicates stronger coordination among traits with specific attributes [64]. Node degree was calculated as the total number of edges adjacent to a focal trait, where those with high degree values were considered “central traits”. Node betweenness was defined as the number of shortest paths between trait pairs that pass through the focal trait [65].

To address concerns regarding multiple testing correction and threshold sensitivity, we evaluated the robustness of our networks. Specifically, to assess the stability of network parameters, a bootstrapping approach was employed by randomly resampling 75% of the traits over 999 iterations to calculate the mean and standard error of each parameter. All trait data were log-transformed before analysis. Statistical analyses and visualization were conducted using R (version4.3.2) and Origin 2021, with the significance level set at *p* < 0.05.

## 5. Conclusions

This study systematically elucidates the driving mechanisms of leaf trait variation and the divergent adaptive strategies of male and female *P. euphratica* under arid conditions, based on variance partitioning and plant trait network analysis. The results demonstrated that nutrient-and stress- responsive traits, including leaf *N*, *K*, and *Pro*, were primarily driven by sex, whereas biomass, photosynthetic, and water-use efficiency traits were mainly governed by developmental stage. The interaction between sex and developmental stage explained a high proportion of the variation in leaf anatomical structures, suggesting that the sexual dimorphism of leaf traits in *P. euphratica* may exhibit ontogenetic contingency, with a trend of phase-specific enhancement during growth. Leaf trait network analysis further revealed significant differences in network topology between the sexes. Male plants constructed a synergistic LTN with high connectivity and short path lengths, wherein physiological traits related to photosynthesis, transpiration, ion regulation, and antioxidant defense were closely interconnected. Based on network topology theory, we speculate that this highly connected structure may facilitate synergistic changes among multiple traits, potentially providing males with stronger physiological regulatory capacity and phenotypic plasticity during short-term environmental fluctuations. In contrast, the LTN of female plants exhibited a higher degree of modularity, where traits related to morphogenesis and biomass allocation (e.g., LA, LDW, SLA) showed higher network centrality. In ecological theory, high modularity is generally considered to enhance buffering between functional modules; this may reflect a potential stability strategy adopted by female plants to balance structural investment and resource allocation amidst reproductive costs.

Along the vertical canopy gradient, the core nodes (traits with high betweenness or centrality) of the LTNs displayed clear spatial shift characteristics. The network cores of male plants in the mid-to-upper canopy were mainly concentrated on stomatal and water transport indicators, suggesting a higher sensitivity to hydraulic limitations and micro-environmental changes in this layer. Meanwhile, the topological changes in female networks were relatively stable along the vertical gradient, with core nodes mostly involving anatomical structures (e.g., FTT) and nutrient metabolism indicators, suggesting a tendency to maintain physiological homeostasis at different heights through the coordination of structure and metabolism. Overall, we speculate that male plants prioritize rapid resource acquisition and dynamic physiological responses, whereas female plants emphasize morphological structural stability and long-term resource conservation.

The divergent adaptive strategies identified here not only deepen our understanding of plant sexual dimorphism but also provide practical guidance for ecological restoration in arid regions. In future forest conservation and plantation management, spatial differences between male and female plants in water use and micro-environmental responses should be fully considered. Rationally optimizing the sex ratio based on site conditions and moisture gradients will enhance the overall stress resistance and ecological stability of arid riparian ecosystems.

## Figures and Tables

**Table 1:** Basic information of female and male samples of *P. euphratica*.

Sex	Diameter Classes	Average Diameter at Breast	Average Tree Height (m)	Average Tree Age (Year)
Female	8	8.33	7.53	8.10
	12	14.30	9.47	9.30
	16	17.67	11.27	10.37
	20	23.23	12.87	11.17
Male	8	9.33	7.97	8.37
	12	14.37	10.00	9.70
	16	17.33	10.93	10.13
	20	24.83	12.70	11.10

## Data Availability

The datasets generated and/or analyzed during the current study are not publicly available as they form part of ongoing research. Access to the data may be granted upon reasonable request, subject to the completion of the associated studies.

## Supplementary Materials

The following supporting information can be downloaded at https://www.mdpi.com/article/10.3390/plants15121770/s1, Figure S1. Degree centrality of network nodes for leaf traits in male and female *P. euphratica* at different tree heights. A: 2 m; B: 4 m; C: 6 m; D: 8 m; E: 10 m; F: 12 m.

## Abbreviation

Leaf Trait	Abbreviation	Leaf Trait	Abbreviation
Leaf length	LL	Leaf water potential	LWP
Leaf width	LW	Instantaneous water use efficiency	WUEi
Leaf index	LI	Chlorophyl	CHL
Leaf area	LA	Carbon Usage Effectiveness	CUE
Leaf thickness	LT	Proline	Pro
Petiole length	PL	Malondialdehyde	MDA
Petiole thickness	PT	Soluble sugar	SS
Leaf fresh weight	LFW	Soluble protein	SP
Leaf dry weight	LDW	Carbon	C
Specific leaf area	SLA	Nitrogen	N
Palisade tissue thickness	FTT	Phosphorus	P
Spongy tissue thickness	STT	Potassium	K
Palisade / spongy ratio	PSR	Degree centrality	DC
Main vein xylem thickness	MXT	Betweenness centrality	BC
Vessel area	CA	Average path length	L
Net photosynthetic rate	Pn	Modularity	Q
Transpiration rate	Tr	edge density	D
Stomatal conductance	Gs	Clustering coefficient	C
Intercellular CO_2_ concentration	Ci	Diameter	Diameter

## Appendix A

### Appendix A.1. Analysis of the Variation Range of Leaf Traits

Comparison of the coefficient of variation (CV) for each trait between male and female plants revealed significant sexual dimorphism in the physiological, morphological, and biochemical traits of *P. euphratica* (Figure A1). The traits exhibiting the greatest variation in male plants were total K content, *Gs*, instantaneous WUE, and MXT (Figure A1B), whereas the degree of variation was smaller for indicators related to basal metabolism and stress resistance, such as *Pn*, MDA, Pro, and total leaf *C*. In contrast to male plants, the traits with the highest variation in female plants were mainly *Gs*, LWP, CHL, and PL (Figure A1A), whereas variation in leaf total C, LT, and *Ci* was relatively low.

### Appendix A.2. Cluster Analysis of Leaf Traits

Based on hierarchical cluster analysis (HCA), the 31 leaf traits of male and female *P. euphratica* were categorized into 7 significant functional modules (Figure A2). These modules correspond to different ecophysiological functional units, reflecting the coordinated trait-regulation network formed by *P. euphratica* during long-term evolution and environmental adaptation. Module I included SLA, LA, LDW, LFW, representing morphological and biomass-related traits, as well as PW, PL, and MDA. Module II was mainly composed of WUE, *Tr*, and CUE. In Module III, *Pn*, *Gs*, and *Ci* wre grouped together with K and Pro. Module IV comprised leaf morphological traits LL, LI, LT and SS, SP. Module V included CHL, LWP, total carbon, nitrogen, and phosphorus contents (C, N, P). Module VI (MXT, CA) and Module VII (FTT, STT, PSR) correspond to traits related to main vein hydraulic capacity and mesophyll structural characteristics, respectively.

Based on the modularization results, 7 representative core traits were further selected from the functional modules to characterize the adaptive strategies of coordinated multi-dimensional trait variations in *P. euphratica*. These 7 core traits were SP, LDW, *Gs*, WUE, LWP, PSR, and MXT. These traits encompass several key functional dimensions, including resource storage and allocation, gas exchange, water status regulation, and leaf anatomy. Such modular characteristics of multi-trait coordination indicate that *P. euphratica* may enhance its adaptability to extreme drought and saline–alkaline environments through the plasticity of morphological and structural traits combined with the regulation of physiological and functional traits.

### Appendix A.3. Comprehensive Trait Structure Analysis

Principal component analysis (PCA) revealed significant sexual dimorphism in the coordinated variation patterns of leaf traits in *P. euphratica* (Figure A3). In male individuals, LWP, SP, and CUE showed high loadings on the first principal component (PC1), whereas MXT and *Gs* contributed more strongly to the second principal component (PC2) (Figure A3B). In female individuals, PC1 was primarily driven by *Gs*, MXT, and CUE, while PC2 exhibited relatively high loadings for SP and LWP, which are associated with water status and nutrient storage (Figure A3A). These results indicate a pronounced divergence in the coordinated structure of key functional traits between male and female plants, potentially reflecting differences in resource-use and adaptive strategies. Male individuals tended to emphasize the coordinated response between metabolic processes and water status, whereas female individuals showed a closer association between structural traits and stomatal regulation.

Furthermore, the trait contribution patterns of male and female *P. euphratica* changed significantly across developmental stages (Figure A3). For male plants, at diameter class 8, SP and PSR contributed more strongly; at diameter class 12, the contribution of LWP increased significantly; at diameter class 16, the contributions of MXT and LDW were enhanced; and by diameter class 20, the contributions of *Gs* and CUE increased again (Figure A3B). In contrast, the changes in trait contributions of female plants across different diameter classes were relatively stable. At diameter class 8, the contributions of MXT, LWP, and SP were high; at diameter class 12, LWP and SP still maintained high contributions, while the contribution of MXT decreased slightly; at diameter class 16, the contributions of CUE and LDW were enhanced; and at diameter class 20, the contributions of Gs and PSR became more prominent. Overall, the trait contribution patterns differed markedly between male and female plants at different developmental stages, indicating that their coordinated trait variations possess significant sex-specific and stage-specific characteristics (Figure A3A).
